# Neoadjuvant Chemotherapy or Endocrine Therapy for Invasive Ductal Carcinoma of the Breast With High Hormone Receptor Positivity and Human Epidermal Growth Factor Receptor 2 Negativity

**DOI:** 10.1001/jamanetworkopen.2021.1785

**Published:** 2021-03-12

**Authors:** Jiaqiang Zhang, Chang-Yun Lu, Ho-Min Chen, Szu-Yuan Wu

**Affiliations:** 1 Department of Anesthesiology and Perioperative Medicine, Henan Provincial People’s Hospital, People’s Hospital of Zhengzhou University, Zhengzhou, Henan, China; 2 Department of General Surgery, Lo-Hsu Medical Foundation, Lotung Poh-Ai Hospital, Yilan, Taiwan; 3 Department of Food Nutrition and Health Biotechnology, Asia University College of Medical and Health Science, Taichung, Taiwan; 4 Big Data Center, Lo-Hsu Medical Foundation, Lotung Poh-Ai Hospital, Yilan, Taiwan; 5 Division of Radiation Oncology, Lo-Hsu Medical Foundation, Lotung Poh-Ai Hospital, Yilan, Taiwan; 6 Department of Healthcare Administration, Asia University College of Medical and Health Science, Taichung, Taiwan; 7 School of Dentistry, College of Oral Medicine, Taipei Medical University, Taipei, Taiwan

## Abstract

**Importance:**

Although neoadjuvant endocrine therapy (NET) is an alternative to chemotherapy for strongly hormone receptor (HR)–positive and human epidermal growth factor receptor 2 (*ERBB2*)–negative breast cancer, evidence is currently lacking regarding the probable survival outcomes of NET in comparison with those of neoadjuvant chemotherapy (NACT) for this cancer.

**Objective:**

To evaluate all-cause mortality among patients with strongly HR-positive and *ERBB2-*negative breast cancer treated with NET vs NACT.

**Design, Setting, and Participants:**

This cohort study included patients with a diagnosis of invasive ductal carcinoma (IDC) with strong HR positivity and *ERBB2 *negativity, treated between January 1, 2009, and December 31, 2016, with follow-up from the index date (ie, date of IDC diagnosis) to December 31, 2018. The data came from the Taiwan Cancer Registry Database. Data were analyzed from January to November 2020.

**Exposures:**

NET vs NACT for IDC with strong HR positivity and ERBB2 negativity.

**Main Outcomes and Measures:**

The primary end point was all-cause mortality. Propensity score matching was performed, and Cox proportional hazard models were used to analyze all-cause mortality among patients undergoing different neoadjuvant treatments.

**Results:**

A total of 640 patients (297 [46.4%] aged 20-49 years) undergoing NET (145 patients [22.7%]) or NACT (495 patients [77.3%]) were eligible for further analysis. In the multivariate Cox regression analyses, the adjusted hazard ratio (aHR) for all-cause mortality among the NET cohort compared with the NACT cohort was 2.67 (95% CI, 1.95-3.51; *P* < .001). The aHRs for age were 1.13 (95% CI, 1.03-2.24), 1.25 (95% CI, 1.13-2.45), and 1.37 (95% CI, 1.17-3.49) for all-cause mortality among patients aged 50 to 59, 60 to 69, and 70 years or older, respectively, compared with those aged 20 to 49 years (*P* = .002); the aHR for all-cause mortality among premenopausal women was 1.35 (95% CI, 1.13-1.56) compared with postmenopausal women (*P* < .001); and that of patients with a Charlson Comorbidity Index score of 2 or greater was 1.77 (1.37-2.26) compared with those with a score of 0 (*P* < .001). The aHRs of all-cause mortality for clinical tumor stage 2, 3, and 4 compared with 1 were 1.84 (95% CI, 1.07-3.40), 1.97 (95% CI, 1.03-3.77), and 2.49 (95% CI, 1.29-4.81), respectively (*P* = .009). The aHRs for all-cause mortality by clinical nodal (cN) stages were 1.49 (95% CI, 1.13-1.99) and 1.84 (95% CI, 1.31-2.61) for cN stage 1 and cN stages 2 or 3, respectively, compared with cN stage 0 (*P* = .005); those for differentiation were 1.77 (95% CI, 1.24-2.54) and 2.31 (95% CI, 1.61-3.34) for differentiation grade 2 and differentiation grade 3, respectively, compared with differentiation grade 1 (*P* < .001).

**Conclusions and Relevance:**

The findings of this study suggest that for patients with strongly HR-positive and *ERBB2*–negative IDC, NACT may be considered the first choice for neoadjuvant treatment.

## Key Points

**Question:**

Is neoadjuvant endocrine therapy (NET) an alternative treatment for neoadjuvant chemotherapy (NACT) in patients with invasive ductal carcinoma (IDC) of the breast who have strong hormone receptor (HR) positivity and human epidermal growth factor receptor 2 (*ERBB2*) negativity?

**Findings:**

This cohort study of 640 patients undergoing NET or NACT found that the adjusted hazard ratios for all-cause mortality in the NET group were significantly higher than in the NACT group.

**Meaning:**

These findings suggest that NACT may be considered the first choice for neoadjuvant treatment for patients with strongly HR-positive and *ERBB2*-negative IDC.

## Teaser

This cohort study evaluate all-cause mortality among patients with strongly hormone receptor–positive and human epidermal growth factor receptor 2 (*ERBB2*)–negative breast cancer treated with neoadjuvant endocrine therapy vs neoadjuvant chemotherapy.

## Introduction

The goal of neoadjuvant therapy is to improve surgical outcomes by inducing tumor shrinkage through effective systemic therapy; neoadjuvant therapy is useful for this because it can be initiated sooner than other therapies and treatment response can be assessed.^1^ The therapy is appropriate for many patients with locally advanced breast cancer (regardless of biologic subtype), which is generally classified as stage III, as well as for the subset of patients with stage IIB cancers and T3 disease.^2,3^ For patients with stage II cancers, either primary surgery or neoadjuvant therapy may be used, with neoadjuvant therapy being appropriate for patients who wish to undergo breast-conserving surgery (BCS) but are not candidates due to a high tumor size–to–breast size ratio.^4,5^ However, hormone receptor (HR)–positive, human epidermal growth factor receptor 2 (*ERBB2*)–negative cancers are less likely to respond to neoadjuvant chemotherapy (NACT) than are other biologic subtypes.^6,7,8,9^ For patients with stage I, HR-positive, *ERBB2*-negative disease, physicians in Taiwan generally prefer to perform primary surgery than to commence neoadjuvant therapy because these patients are likely to have favorable surgical outcomes.

Neoadjuvant or presurgical therapy refers to therapy administered before surgery. It has been used for more than 2 decades to downstage locally advanced and unresectable primary breast cancers to make them operable.^10,11^ Several studies, including the landmark National Surgical Adjuvant Breast and Bowel Project 18 trial, have demonstrated that the administration of the same chemotherapy in the neoadjuvant setting and the adjuvant setting is associated with similar outcomes.^12,13,14^ Although NACT is conventionally used to downstage locally advanced and unresectable primary breast cancers, numerous studies have identified neoadjuvant endocrine therapy (NET) as an alternative to chemotherapy for strongly HR-positive and *ERBB2*-negative tumors.^15,16,17,18,19^

In this study, we focused on the outcomes of neoadjuvant treatment for strongly HR-positive and *ERBB2*-negative invasive ductal carcinoma (IDC) of the breast. The survival outcomes of NACT and NET were evaluated for patients with strongly HR-positive and *ERBB2*-negative IDC who might benefit from NET.

## Methods

We established a cohort comprising female patients with IDC by using data from the Taiwan Cancer Registry database (TCRD), which is maintained by the Collaboration Center of Health Information Application. Our protocols were reviewed and approved by the institutional review board of Taipei Medical University. Informed consent was waived because the data sets are covered under the Personal Information Protection Act. We enrolled patients who received an IDC diagnosis between January 1, 2009, and December 31, 2016, and underwent NACT or NET followed by mastectomy. The follow-up duration was from the index date to December 31, 2018. The index date was the date of IDC diagnosis. The median (interquartile range) follow-up time was 60.6 (47.2-73.9) months for the NACT group and 55.4 (41.8-69.7) months for the NET group. The TCRD contains detailed cancer-related information of patients, including the clinical stage (according to the American Joint Committee on Cancer [AJCC], seventh edition), treatment modalities, pathologic data (including pathologic stage), irradiation doses, HR status, *ERBB2* status, and chemotherapy regimens used.^20,21,22,23,24,25,26,27,28^ Patient diagnoses were confirmed on the basis of pathologic data, and patients who received a new diagnosis of IDC were confirmed to have no other cancers. The NACT regimen applied in this study consisted of at least 4 cycles of anthracycline-based or taxane-based regimens or both every 3 weeks, and the NET regimen consisted of aromatase inhibitors (AIs) for postmenopausal women and tamoxifen or AIs combined with the gonadotropin-releasing hormone analog for premenopausal women every 4 weeks for at least 4 months. For those in the NET group with residual disease who were able to proceed with surgery and had no contraindications for chemotherapy, we followed the same decision-making process for adjuvant chemotherapy as that for patients who did not receive neoadjuvant treatment. Nevertheless, no adjuvant chemotherapy was administered to patients in the NET group. Other inclusion criteria were being aged 20 years or older and having AJCC stage IIB to IIIC disease. Patients with metastasis, missing sex data, age younger than 20 years, nonstandard adjuvant breast radiation therapy (RT; vs standard adjuvant RT, consisting of irradiation to the chest wall, whole breast, and regional nodes with a minimum of 50 Gy), unclear differentiation of tumor grade, unclear pathologic response, missing HR status, missing *ERBB2* status, or unclear staging were excluded.

Furthermore, we excluded patients with an unclear NACT regimen, nonanthracycline-based or nontaxane-based regimens, fewer than 4 cycles of NACT, less than 4 months of NET, non-AI–based or nontamoxifen-based NET regimens, ill-defined nodal surgery, or nonrecorded hospital type^29^ (academic center or community hospital) from our cohort. Pathologic responses were categorized into upstaging (an increase from the clinical stage to an advanced pathologic stage), equal stages (clinical stage equal to pathologic stage), downstaging (a decrease in the clinical stage to an earlier pathologic stage), and pathologic complete response (pCR, absence of residual invasive cancer). According to the results of the Peri-Operative Endocrine Therapy–Individualizing Care (POETIC),^33^ American College of Surgeons Oncology Group (ACOSOG) Z1031,^30^ and ALTERNATE^32^ trials and other observational studies,^2,18^ NET is an acceptable alternative option for those with *ERBB2*-negative tumors that are strongly HR positive. Tumors that are more likely to respond to NET have strong HR expression (eg, ≥50% staining for the estrogen receptor [ER] or an Allred score of 6 to 8); by contrast, progesterone receptor is not a strong indicator for NET in breast IDC, according to previous studies.^2,18,30,31^ Strong HR positivity was defined in our study as at least 50% positive nuclear staining through immunohistochemistry for the ER with an Allred score of 6 to 8,^2,18,30,31,32,33,34^ and *ERBB2* negativity^2,18^ was defined as an immunohistochemistry score of less than 3 or a fluorescence in situ hybridization ratio of 2 or greater.^29,35^ Progesterone receptor status was not a criterion; progesterone receptor–positive or negative nuclear staining through immunohistochemistry for the progesterone receptor could be included in our population. In total, 4419 patients were excluded from the study.

Finally, we enrolled patients with strongly HR-positive and *ERBB2*-negative IDC receiving neoadjuvant treatments followed by mastectomy and grouped them based on whether they received NACT or NET. Comorbidities were assessed using the Charlson Comorbidity Index (CCI).^36,37^ The CCI has prognostic significance for all-cause death in patients with breast cancer.^38,39^ Only comorbidities observed 6 months before the index date were included, and new onset comorbidities, which happened within 6 months before the index date, were excluded. This criterion means we could analyze the association of long-term comorbidities with survival for our patients. Comorbidities were identified according to the primary *International Classification of Diseases, Ninth Revision, Clinical Modification* (*ICD-9-CM*) codes; diseases present at first admission and those identified more than twice during outpatient visits were included as comorbidities.

### Statistical Analysis

After adjustment for confounders, a Cox proportional hazard model was established to model the time from the index date to all-cause mortality for these patients with IDC. To reduce the effects of potential confounders when therapy outcomes between groups were compared, propensity score matching (PSM) was performed. The matching variables used were age (ie, 20-49, 50-59, 60-69, and ≥70 years), menopausal status, diagnosis year, CCI score, differentiation, clinical tumor (cT) stage, clinical nodal (cN) stage, hospital level, surgical type, adjuvant RT, and nodal surgery. In Taiwan, hospitals are classified in various levels, such as academic hospitals (termed medical centers) with high volume and nonacademic hospitals (nonmedical centers) with low volume. Some studies have found that the survival rate of patients with cancer varies by hospital level.^40,41,42^ We matched the cohorts on the logit of the propensity score by using calipers with widths equal to 0.2 of the standard deviation of the logit of the propensity score.^43^ Matching is a common technique for selecting a control group with identical background covariates as study participants to minimize differences between individuals that the investigator believes must be controlled. A Cox model was used to regress survival on treatment status, with a robust sandwich estimator used to account for the clustering within matched sets.^44^ Multivariate Cox regression analyses were performed to calculate hazard ratios (HRs) to determine whether factors such as age, menopausal status, year of diagnosis, CCI score, differentiation, cT stage, cN stage, or hospital level were independently associated with treatment choice. Potential factors associated with treatment choice were controlled for in the analysis (Table 1), and all-cause mortality was the end point in both groups.

**Table 1.** Characteristics of Patients With Strongly Hormone Receptor–Positive and Human Epidermal Growth Factor Receptor 2–Negative Invasive Ductal Carcinoma Receiving NACT or NET, After Propensity Score Matching

Characteristic	Patients by treatment group, No. (%)		*P* value
	NACT (n = 495)	NET (n = 145)	
Age, y			
20-49	230 (46.5)	67 (46.2)	.99^a^
50-59	156 (31.5)	45 (31.0)	
60-69	72 (14.5)	21 (14.7)	
≥70	37 (7.5)	12 (8.3)	
Menopausal status			
Postmenopausal	250 (50.5)	64 (44.1)	.89^a^
Premenopausal	245 (49.5)	81 (55.9)	
Year of diagnosis			
2009-2012	205 (41.4)	57 (39.3)	.46^a^
2013-2016	290 (58.6)	88 (60.7)	
CCI score			
0	405 (81.8)	107 (73.8)	.89^a^
1	58 (11.7)	22 (15.2)	
≥2	32 (6.5)	16 (11.0)	
Differentiation grade			.
1	69 (14.0)	27 (18.6)	.70^a^
2	357 (72.0)	95 (65.5)	
3	69 (14.0)	23 (15.9)	
cT stage			
1	42 (8.5)	10 (6.9)	.18^a^
2	328 (66.3)	93 (64.1)	
3	62 (12.5)	16 (11.0)	
4	63 (12.7)	26 (17.9)	
cN stage			
0	254 (51.3)	83 (57.2)	.15^a^
1	191 (38.6)	49 (33.8)	
2	33 (6.7)	7 (4.8)	
3	17 (3.4)	6 (4.1)	
AJCC clinical stage			
IIA	265 (53.5)	78 (53.8)	.99^a^
IIB	104 (21.0)	28 (19.3)	
III	126 (25.5)	39 (26.9)	
Hospital level			
Academic	330 (66.7)	98 (67.6)	.75^a^
Nonacademic	165 (33.3)	47 (32.4)	

Abbreviations: AJCC, American Joint Committee on Cancer; CCI, Charlson Comorbidity Index; cN, clinical nodal; cT, clinical tumor; NACT, neoadjuvant chemotherapy; NET, neoadjuvant endocrine therapy.

^a^ *P* value was estimated using the χ^2^ test.

Cox proportional hazard curves were used to estimate all-cause mortality (specifically, overall survival [OS]) in patients receiving either treatment. Covariates in the NET group were 1:4, 1:3, 1:2, or 1:1 matched to those in the NACT group through PSM with replacement, and all matched covariates in the NET and NACT groups were included in the Cox proportional hazards model. After adjustment for confounders, the Cox proportional hazards method was used to model the time from the index date to all-cause mortality. In the multivariate analysis, HRs were adjusted for age, menopausal status, diagnosis year, CCI score, differentiation, cT stage, cN stage, hospital level, surgical type, adjuvant RT, nodal surgery, and pathologic stage. All analyses were performed using SAS version 9.3 (SAS Institute). In a 2-tailed Wald test, *P* < .05 was considered significant.

OS was estimated using the Kaplan-Meier method. Differences between the neoadjuvant treatment modalities were determined using the stratified log-rank test to compare survival curves (stratified according to matched sets).^45^

## Results

The matching process yielded a final cohort of 640 patients (297 [46.4%] aged 20-49 years; 495 [77.3%] in the NACT group; 145 [22.7%] in the NET group) eligible for further analysis; their characteristics are summarized in Table 1. Most patients had a CCI score of 0 (512 [80.0%]) or 1 (80 [12.5%]). Age distribution was balanced between the 2 groups (Table 1). Menopausal status, year of diagnosis, CCI score, differentiation, cT stage, cN stage, hospital level, surgical type, adjuvant RT, and nodal surgery were similar in the 2 cohorts, and no statistically significant differences were present in the variables of the 2 cohorts. Postneoadjuvant treatment response, namely pathologic stage, pathologic response (pCR, downstage, equal stage, or upstage), or death, were not matched because the survival time and postneoadjuvant treatment responses were inconsistent between the 2 groups (eTable in the Supplement). The outcomes of patients with strongly HR-positive and *ERBB2*-negative IDC receiving neoadjuvant treatments varied significantly. Pathologic responses were significantly more favorable in the NACT group than in the NET group (eg, pCR: 67 [12.5%] vs 9 [6.2%]; *P* < .001; downstaging: 172 [34.7%] vs 29 [20.0%]; *P* < .001) (eTable in the Supplement). Moreover, although clinical stages, including cT stage and cN stage, were similar between the groups, pathologic AJCC stages were significantly more advanced in the NET group than the NACT group (eg, stage III: 50 [34.5%] vs 124 [25.1%]; *P* < .001), indicating poor pathologic responses after NET. The death rate was higher in the NET group than in the NACT group (38 [26.2%] vs 71 [14.3%]; *P* = .003) (eTable in the Supplement). However, no significant differences were observed in the BCS rates of the NACT and NET groups (198 [40.0%] vs 55 [37.9%]) .

The multivariate Cox regression analyses indicated that NACT was associated with a higher OS than was NET. The HRs in the univariate analysis were similar to those in the multivariate Cox regression analysis. No significant differences were observed in the explanatory variables before neoadjuvant treatments, except for neoadjuvant treatment, age, menopausal status, CCI score, cT stage, cN stage, and differentiation (Table 2). In the multivariate Cox regression analyses, the adjusted HR (aHR) for NET compared with NACT was 2.67 (95% CI, 1.95-3.51; *P* < .001). The aHRs for age were 1.13 (95% CI, 1.03-2.24), 1.25 (95% CI, 1.13-2.45), and 1.37 (95% CI, 1.17-3.49) for all-cause mortality for patients aged 50 to 59 years, 60 to 69 years, and 70 years or older, respectively, compared with those aged 20 to 49 years (*P* = .002). The aHR for premenopausal status was 1.35 (95% CI, 1.13-1.56) compared with postmenopausal status (*P* < .001); that of CCI score of 2 or greater was 1.77 (95% CI, 1.37-2.26) compared with a CCI score of 0 (*P* < .001). The aHRs of cT stage 2, 3, and 4 compared with 1 were 1.84 (95% CI, 1.07-3.40), 1.97 (95% CI, 1.03-3.77), and 2.49 (95% CI, 1.29-4.81), respectively (*P* = .009). The aHRs for cN stages were 1.49 (95% CI, 1.13-1.99) and 1.84 (95% CI, 1.31-2.61) for cN stage 1 and cN stage 2 or 3, respectively, compared with 0 (*P* = .005); those of differentiation were 1.77 (95% CI, 1.24-2.54) and 2.31 (95% CI, 1.61-3.34) for all-cause mortality for differentiation stage 2 and differentiation stage 3, respectively, compared with differentiation stage 1 (*P* < .001). The aHRs of pathologic stages were 1.17 (95% CI, 1.06-2.54), 1.25 (95% CI, 1.12-2.82), and 2.17 (95% CI, 1.30-3.60) for all-cause mortality for AJCC pathologic stages I, II, and III, respectively, compared with pathologic stage T0N0 (*P* < .001) (Table 2).

**Table 2.** Multivariable Analysis of All-Cause Mortality Among Patients With Strongly Hormone Receptor–Positive and Human Epidermal Growth Factor Receptor 2–Negative Invasive Ductal Carcinoma Receiving Neoadjuvant Treatments

Factor	All-cause mortality	
	aHR (95% CI)	*P* value
Neoadjuvant treatment		
NACT	1 [Reference]	<.001
NET	2.67 (1.95-3.51)	
Age, y		
20-49	1 [Reference]	.002
50-59	1.13 (1.03-2.24)	
60-69	1.25 (1.13-2.45)	
≥70	1.37 (1.17-3.49)	
Menopausal status		
Postmenopausal	1 [Reference]	<.001
Premenopausal	1.35 (1.13-1.56)	
Year of diagnosis		
2009-2012	1 [Reference]	.17
2013-2016	1.17 (0.88-1.43)	
CCI score		
0	[Reference]	<.001
1	1.08 (0.87-1.35)	
≥2	1.77 (1.37-2.26)	
cT stage		
1	1 [Reference]	.009
2	1.84 (1.07-3.40)	
3	1.97 (1.03-3.77)	
4	2.49 (1.29-4.81)	
cN stage		
0	1 [Reference]	.005
1	1.49 (1.13-1.99)	
2-3	1.84 (1.31-2.61)	
Differentiation grade		
1	1 [Reference]	<.001
2	1.77 (1.24-2.54)	
3	2.31 (1.61-3.34)	
Surgical type		
BCS	1 [Reference]	.32
TM	1.17 (0.70-1.44)	
Nodal surgery		
ALND	1 [Reference]	.26
SLNB	0.77 (0.60-1.27)	
Adjuvant RT	1.44 (0.69-1.81)	.34
Hospital level		
Academic	1 [Reference]	.78
Nonacademic	0.97 (0.82-1.16)	
Pathologic stage		
T0N0	1 [Reference]	<.001
I	1.17 (1.06-2.54)	
II	1.25 (1.12-2.82)	
III	2.17 (1.30-3.60)	

Abbreviations: aHR, adjusted hazard ratio; ALND, axillary lymph node dissection; BCS, breast-conserving surgery; CCI, Charlson Comorbidity Index; cN, clinical nodal; cT, clinical tumor; NACT, neoadjuvant chemotherapy; NET, neoadjuvant endocrine therapy; RT, radiation therapy; SLNB, sentinel lymph node biopsy; TM, total mastectomy.

The Figure presents the survival curves for all-cause mortality, obtained using the Kaplan-Meier method, for the PSM cohort of patients with strongly HR-positive and *ERBB2*-negative IDC receiving neoadjuvant treatments. The OS rate for NACT was higher than that for NET for all patients (*P* < .001).

**Figure.**
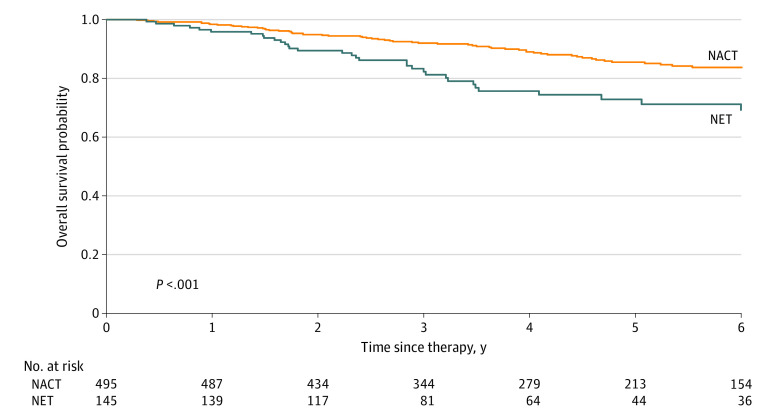
Kaplan-Meier Survival Curve for Overall Survival in Patients With Strongly Hormone Receptor–Positive and Human Epidermal Growth Factor Receptor 2–Negative Invasive Ductal Carcinoma Receiving Neoadjuvant Treatments NACT indicates neoadjuvant chemotherapy; NET, neoadjuvant endocrine therapy.

## Discussion

The choice of neoadjuvant treatment is informed by studies in the adjuvant setting that have assessed OS as the outcome of interest as well as by studies in the neoadjuvant setting that have assessed pathologic response rates and BCS rates as the outcomes of interest.^46,47,48^ Typically, the choice of therapy is NACT followed by surgery, NET followed by surgery, or upfront surgery with adjuvant chemotherapy, if indicated.^16,17,46,47,48^ All patients with HR-positive cancers should receive endocrine therapy in the adjuvant setting.^49^ For most medically fit patients requiring neoadjuvant treatment, chemotherapy is administered in accordance with the robust literature documenting associated response rates and survival benefits.^2^ However, for those with *ERBB2*-negative tumors that are strongly HR positive, NET may be an acceptable alternative.^15^ Breast cancers that are more likely to respond to NET have strong HR expression.^15,16,17,18,19^ For patients with HR-positive, *ERBB2*-negative disease who are not candidates for chemotherapy due to significant comorbidities or disease severity, options include upfront surgery or NET, which can enable tumor volume reduction prior to resection under local anesthesia, possibly leading to a need for less extensive surgery.^15,16,17,18,19^ Taken together, study results have indicated that NET is a feasible treatment option for patients with IDC with strongly HR-positive and *ERBB2*-negative expression. However, to our knowledge, no head-to-head study has compared the association of NACT and NET with outcomes in patients with strongly HR-positive and *ERBB2*-negative IDC. Therefore, we conducted this PSM-based study to estimate the OS of these patients receiving NACT or NET.

Chemotherapy can shrink HR-positive breast tumors and make more favorable surgical options available, but it is less likely to achieve pCR in HR-positive breast cancers, especially luminal A cancers, than in more proliferative histologies.^2,50^ Among those with HR-positive, *ERBB2*-negative disease, the percentage of patients with pCR after NACT was reported to be between 8% and 16%, depending on tumor differentiation,^16,17^ which is compatible with our identified rate (12.5%) (eTable in the Supplement). Previous studies on NET for premenopausal women have been limited to phase 2 studies,^18,19^ but they have suggested worse response rates for NET relative to NACT.^18^ The results of the subgroup analysis in the Grupo Español de Investigación del Cáncer de Mama (GEICAM) study are compatible with our findings; the pathologic response rate for NACT was inferior to that for NET (pCR and downstaged cancer: 47.2% vs 26.2%) (eTable in the Supplement).^18^ However, our definition of pathologic response rates based on AJCC stages differed from that in the GEICAM study; moreover, both premenopausal and postmenopausal women were included in our study. Other available data for postmenopausal women suggest that NET is associated with similar rates of BCS as NACT, with lower toxic effects, although survival data for NET are not yet available.^18,51,52,53^ In our study, the BCS rate for NET was compatible with rates in previous studies, and no significant difference was observed compared with NACT.^18,51,52,53^

As Table 1 suggests, the covariates in our PSM cohorts were balanced, and no significant differences were observed between the NACT and NET cohorts. Postneoadjuvant treatment responses, including pathologic response and pathologic stage, and the BCS rate were not matched in our PSM cohorts (eTable in the Supplement). As shown in the eTable in the Supplement, a poor pathologic response was observed in the NET group compared with the NACT group; this finding is compatible with that of a previous study.^18^ In addition, pathologic stages were more advanced in the NET group than in the NACT group (eTable in the Supplement); the initial cT and cN stages were well matched and exhibited no differences in our PSM cohorts (Table 1). According to previous studies, pathologic responses and pathologic stages after neoadjuvant treatments are significant prognostic factors for OS, especially pCR.^2,54,55^ Therefore, the survival rate in our NACT group, with its more favorable pathologic responses and earlier pathologic stages, was superior to that of the NET group; this finding is also compatible with reported findings.^54,55^ The novelty of our study is its investigation of whether NET is an alternative neoadjuvant treatment for NACT in strongly HR-positive and *ERBB2*-negative breast IDC. The survival rate appeared to be inferior in the NET group (eTable in the Supplement). To our knowledge, this study is also the leading study to report the rates of pCR, downstaging, equal staging, and upstaging in patients with strongly HR-positive and *ERBB2*-negative IDC who received sufficient-duration NACT or NET. To our knowledge, no study had previously examined the rate of BCS or pathologic responses in this patient population. The present study revealed similar pCR outcomes (12.5%) after NACT, compatible with previous findings (13%-26%) for *ERBB2*-negative breast cancer.^14,56^

The results of the multivariable analysis of all-cause death for patients with strongly HR-positive and ERBB2-negative IDC receiving neoadjuvant treatment are presented in Table 2. To our knowledge, this is the leading study in determining the prognostic factors for OS in this patient population. Although NET is accepted as an alternative treatment for *ERBB2*-negative breast cancers that are strongly HR positive, no study has previously compared the association with OS in NACT and NET groups.^15,18,51,52,53^ Our findings indicate that compared with the NACT group, the NET group had a higher risk of all-cause death with an aHR of 2.67 (95% CI, 1.95-3.51) (Table 2). Our outcomes suggest a similar BCS rate between NACT and NET, which is compatible with previous results,^18,51,52,53^ but inferior survival was found compared with the NACT group, even among patients with strongly HR-positive and *ERBB2*-negative breast IDC sensitive to NET.^26,27^ In multivariable analysis, the preneoadjuvant treatment factors of older age, premenopausal status, CCI score of 2 or greater, advanced cT stage (2-4), advanced cN stage (1-3), and poor differentiation (grade 2 or 3) were independent poor prognostic factors for OS. This finding is compatible with previous findings.^54,55^ No evidence had previously indicated that menopausal status is a prognostic factor of OS in strongly HR-positive and *ERBB2*-negative breast IDC. To our knowledge, the current study is the leading study to demonstrate that premenopausal status is an independent poor prognostic factor in OS of strongly HR-positive and *ERBB2*-negative IDC (Table 2).

Kaplan-Meier survival curves of OS in patients with strongly HR-positive and *ERBB2*-negative IDC receiving neoadjuvant treatments after PSM indicated that the NACT group had superior outcomes than the NET group. Moreover, our patients had fewer comorbidities (CCI score of 0-1 for more than 90% of the cohort) (Table 1). Our outcomes might be difficult to extrapolate to patients with multiple comorbidities (ie, CCI score, ≥2) receiving NACT or NET.

The study results suggest that neoadjuvant therapy may be indicated for women with relatively large tumors or locally advanced breast cancer. According to our results, in such situations, patients should receive NACT rather than NET (Table 2). If a premenopausal woman refuses (or is an unfavorable candidate for) NACT, physicians are suggested to proceed to surgical treatment, if possible, rather than attempt NET (Table 2). For patients who are concerned about the extent of definitive surgical treatment, NET may be offered, but patients should be advised that the data in this setting suggest superior pathologic responses, less residual tumor burden, and more favorable survival outcomes with chemotherapy than endocrine therapy (Table 2; eTable in the Supplement). Most women for whom neoadjuvant treatment is indicated receive chemotherapy, although endocrine therapy may be offered as an alternative for some women with strongly HR-positive and *ERBB2*-negative cancer. However, based on our results, we do not suggest NET, especially for healthy patients with relatively few comorbidities (Table 2).

### Strengths and Limitations

The strengths of this study are its large sample size and the homogeneity of its IDC population. The study included homogenous breast cancer biologic subtypes and pathology (all IDC), similar clinical stages, and homogenous doses and durations of NACT and NET. Most major covariates, such as age, menopausal status, year of diagnosis, CCI score, differentiation, cT stage, cN stage, hospital level, adjuvant RT, and nodal surgery, were included in PSM analysis. To our knowledge, this is the leading and largest head-to-head PSM study to investigate the association of NACT or NET with outcomes among patients with strongly HR-positive and *ERBB2*-negative breast IDC. According to our findings, NACT for strongly HR-positive and *ERBB2*-negative breast IDC is associated with better OS. This finding should be considered in clinical practice and in future prospective clinical trials.

The study has some limitations. First, because all patients were enrolled from an Asian population, the corresponding ethnic susceptibility remains unclear; therefore, our results should be extrapolated to non-Asian populations with caution. Second, the diagnoses of all comorbid conditions were based on *ICD-9-CM* codes. The Taiwan Cancer Registry Administration randomly reviews medical records and interviews patients to verify the accuracy of diagnoses, and hospitals with outlier charges or practices may be audited and heavily penalized if malpractice or discrepancies are identified. However, to obtain crucial information on population specificity and disease occurrence, a large-scale randomized clinical trial comparing carefully selected patients undergoing suitable treatments is essential. Third, patients with a CCI score 2 or greater accounted for less than 10% of the sample after PSM. The superior survival benefits of NACT relative to NET were observed in patients with relatively few comorbidities; thus, our main finding is difficult to extrapolate to patients with substantial comorbidities. Fourth, a central question remains regarding why some patients were offered NET by their physicians. Nevertheless, we made every attempt to propensity match and made adjustments in the multivariable Cox analysis with the aim of making the findings of the observational study as reliable as those of a randomized clinical trial. In fact, designing a randomized clinical trial to confirm whether such neoadjuvant treatments as those studied in this article are appropriate for our patient population would be difficult. This is a clinical study with high value for clinical practice. Fifth, the TCRD does not contain information regarding dietary habits, socioeconomic status, or body mass index, all of which may be potential risk factors for morbidity and might increase the risk of all-cause mortality. However, CCI score was adjusted through PSM. As a result, considering the magnitude and statistical significance of the observed associations in this study, these limitations are unlikely to have affected the conclusions.

## Conclusions

This cohort study evaluated the outcomes of strongly HR-positive and *ERBB2*-negative IDC treated with NACT vs NET. The results suggest that NACT is preferable to NET for neoadjuvant treatment.

## Notes

Supplement. **eTable.** Outcomes of Patients With Strongly Hormone Receptor–Positive and Human Epidermal Growth Factor Receptor 2–Negative Invasive Ductal Carcinoma Receiving Neoadjuvant Treatments After Propensity Score Matching
