# Supplementary material for: Neoadjuvant Chemotherapy or Endocrine Therapy for Invasive Ductal Carcinoma of the Breast With High Hormone Receptor Positivity and Human Epidermal Growth Factor Receptor 2 Negativity
Source: JAMA Netw Open. 2021 Mar 12;4(3):e211785. doi: 10.1001/jamanetworkopen.2021.1785 (PMC7955271; doi:10.1001/jamanetworkopen.2021.1785)
Supplement: Supplement. — eTable. Outcomes of Patients With Strongly Hormone Receptor–Positive and Human Epidermal Growth Factor Receptor 2–Negative Invasive Ductal Carcinoma Receiving Neoadjuvant Treatments After Propensity Score Matching [file jamanetwopen-e211785-s001.pdf]

Supplemental Online Content

Zhang J, Lu CY, Chen HM, Wu SY. Neoadjuvant chemotherapy or endocrine therapy for invasive ductal carcinoma of the breast with high hormone receptor positivity and human epidermal growth factor receptor 2 negativity. *JAMA Netw Open*. 2021;4(3):e211785. doi:10.1001/jamanetworkopen.2021.1785

eTable. Outcomes of Patients With Strongly Hormone Receptor–Positive and Human Epidermal Growth Factor Receptor 2–Negative Invasive Ductal Carcinoma Receiving Neoadjuvant Treatments After Propensity Score Matching

This supplemental material has been provided by the authors to give readers additional information about their work.

© 2021 Zhang J et al. *JAMA Network Open.*

eTable. Outcomes of Patients With Strongly Hormone Receptor–Positive and Human Epidermal Growth Factor Receptor 2–Negative Invasive Ductal Carcinoma Receiving Neoadjuvant Treatments After Propensity Score Matching

		NACT N = 495		NET N = 145		
Variable		n	(%)	n	(%)	*P* value
Pathologic response	pCR	67	(12.5)	9	(6.2)	<0.0001#
	Downstaging	172	(34.7)	29	(20.0)	
	Equal stage	146	(29.5)	59	(40.7)	
	Upstaging	110	(22.2)	48	(33.1)	
Pathologic AJCC stage	ypT0N0	67	(12.5)	9	(6.2)	<0.0001#
	I	85	(17.2)	10	(6.9)	
	II	219	(44.2)	76	(52.4)	
	III	124	(25.1)	50	(34.5)	
Surgical type	BCS	198	(40.0)	55	(37.9)	0.9953#
	TM	297	(60.0)	90	(62.1)	
Nodal surgery	ALND	407	(82.2)	118	(81.4)	0.6530#
	SLNB	88	(17.8)	27	(18.6)	
Adjuvant RT		384	(77.6)	99	(68.3)	0.0536#
Death		71	(14.3)	38	(26.2)	0.0025#

NACT, neoadjuvant chemotherapy; NET, neoadjuvant endocrine therapy; AJCC, American Joint Committee on Cancer; pCR, pathologic complete response; ref, reference group; yp, postneoadjuvant treatment pathologic stage; BCS, breast-conserving surgery; TM, total mastectomy; ALND, axillary lymph node dissection; SLNB, sentinel lymph node biopsy.

#*P* value was estimated using the chi-square test; †*P* value was estimated using analysis of variance.

© 2021 Zhang J et al. *JAMA Network Open.*
